# Immune Cell Confrontation in the Papillary Thyroid Carcinoma Microenvironment

**DOI:** 10.3389/fendo.2020.570604

**Published:** 2020-10-22

**Authors:** Zhenyu Xie, Xin Li, Yuzhen He, Song Wu, Shiyue Wang, Jianjian Sun, Yuchen He, Yu Lun, Jian Zhang

**Affiliations:** Department of Vascular and Thyroid Surgery, The First Hospital, China Medical University, Shenyang, China

**Keywords:** ****papillary thyroid carcinoma, immune cell infiltration, tumor microenvironment, immune escape, TCGA, CIBERSORT

## Abstract

**Background:**

Papillary thyroid cancer has been associated with chronic inflammation. A systematic understanding of immune cell infiltration in PTC is essential for subsequent immune research and new diagnostic and therapeutic strategies.

**Methods:**

Three different algorithms, single-sample gene set enrichment analysis (ssGSEA), immune cell marker and CIBERSORT, were used to evaluate immune cell infiltration levels (abundance and proportion) in 10 data sets (The Cancer Genome Atlas [TCGA], GSE3467, GSE3678, GSE5364, GSE27155, GSE33630, GSE50901, GSE53157, GSE58545, and GSE60542; a total of 799 PTC and 194 normal thyroid samples). Consensus unsupervised clustering divided PTC patients into low-immunity and high-immunity groups. Weighted gene coexpression network analysis (WGCNA) and gene set enrichment analysis (GSEA) were used to analyze the potential mechanisms causing differences in the immune response.

**Results:**

Compared with normal tissues, PTC tissues had a higher overall immune level and higher abundance levels and proportions of M2 macrophages, Tregs, monocytes, neutrophils, dendritic cells (DCs), mast cells (MCs), and M0 macrophages. Compared with early PTC, advanced PTC showed higher immune infiltration and higher abundance levels and proportions of M2 macrophages, Tregs, monocytes, neutrophils, DCs, MCs, and M0 macrophages. Compared to the low-immunity group, the high-immunity group exhibited more advanced stages, larger tumor sizes, greater lymph node metastases, higher tall-cell PTCs, lower follicular PTC proportions, more BRAF mutations, and fewer RAS mutations. Epstein-Barr virus (EBV) infection was the most significantly enriched Kyoto Encyclopedia of Genes and Genomes (KEGG) pathway for key module genes.

**Conclusions:**

In human PTC, M2 macrophages, Tregs, monocytes, neutrophils, DCs, MCs, and M0 macrophages appear to play a tumor-promoting role, while M1 macrophages, CD8+ T cells, B cells, NK cells, and T follicular helper (T_FH_) cells (including eosinophils, γδ T cells, and Th17 cells with weak supporting evidence) appear to play an antitumor role. During the occurrence and development of PTC, the overall immune level was increased, and the abundance and proportion of tumor-promoting immune cells were significantly increased, indicating that immune escape had been aggravated. Finally, we speculate that EBV may play an important role in changing the immune microenvironment of PTC tumors.

## Introduction

Thyroid cancer (TC) is the fifth most common cancer in women in the USA, with an estimated 52,890 new TC cases nationwide in 2020 (1). The incidence of TC has been increasing in recent decades, and showing a trend of rejuvenation (2–7). TC also has the highest rate of growth among diagnosed tumors in the USA (8). Papillary thyroid carcinoma (PTC) belongs to differentiated thyroid carcinoma (DTC) and accounts for approximately 80–85% of all TCs (2, 9). Although the vast majority of PTCs can be cured by surgery and ^131^I treatment, 10% of patients still die from poorly differentiated and advanced tumors (10). A study with a median follow-up period of 27 years also showed that 28% of PTC patients relapsed and 9% died of PTC (11). The reason is partly because the interaction and collective effect of tumors with the surrounding immune microenvironment during the occurrence and development of the tumor promotes immune tolerance and then progresses to immune escape, resulting in the body’s inability to completely clear the tumor.

The immune editing hypothesis was proposed by Dunn in 2002 and divided the interaction between the immune system and the tumor into three stages: (1) elimination: tumor cells are cleared by the immune system before a clinical diagnosis is established. This stage is also called immune surveillance; (2) equilibrium: tumor cells with less immunogenicity gradually replace tumor cells with more immunogenicity; and (3) escape: tumor cells eventually escape the body’s immune surveillance (12). According to this hypothesis, the immune editing of tumors also sculpts the tumor’s immune phenotype while clearing the tumor, enabling the host to generate immune activity against the tumor. When the tumor is clinically diagnosed, it can not only tolerate the immune response but also manipulate the immune system to promote tumor progression. Poschke et al. (13) believe that tumors have two different methods of immune escape: camouflage and sabotage. Camouflage refers to the malformation and loss of the major histocompatibility complex class I (MHC-I) molecules on the surfaces of tumors, allowing tumors to escape the detection of the immune system. Sabotage refers to the ability of some tumors, including PTC, to manipulate part of the immune system to fight against the body’s immune response to protect themselves. In this process, tumors attract and even mediate some immune cells, such as tumor-associated macrophages (TAMs), myeloid-derived suppressor cells (MDSCs), Tregs, and mast cells (MCs). In general, MDSCs, Tregs, and MCs respond to uncontrolled inflammatory reactions in the body, but in tumor progression, they have created an immune microenvironment that allows this process. Existing evidence shows that Tregs, neutrophils, dendritic cells (DCs), MCs, and TAMs play a tumorigenic role in the PTC tumor microenvironment (TME), while CD8+ T cells, B cells, and NK cells play a protective role (14–16). However, the infiltration and role of some major immune cells in PTC are still controversial. Studies on nonprimary immune cells are scarce, and systematic research on the PTC immune microenvironment remains to be conducted. Therefore, accurate assessment of the specific pattern of immune cells in PTC, including not only its phenotype but also its function, is essential for subsequent immune research and new diagnostic and therapeutic strategies.

The purpose of this study was to systematically understand immune cell infiltration in the PTC microenvironment and to summarize the changes in the immune microenvironment during the occurrence and development of PTC. Our research found that M2 macrophages, Tregs, monocytes, neutrophils, DCs, MCs, and M0 macrophages play a tumor-promoting role, while M1 macrophages, CD8+ T cells, B cells, NK cells, and T follicular helper (T_FH_) cells play an antitumor role. During the occurrence and development of PTC, the overall immune level increased, and the abundance and proportion of tumor-promoting immune cells increased significantly. Our research will help researchers systematically understand PTC immune cell infiltration and provide a reference for subsequent basic and clinical research related to PTC immunity.

## Materials and Methods

### Materials

The RNA-seq data (level-three, FPKM) of the thyroid carcinoma (THCA) dataset were downloaded from The Cancer Genome Atlas (TCGA) (https://portal.gdc.cancer.gov/) and included 58 normal thyroid samples (N) and 512 PTC samples (T). The clinical data of THCA were downloaded from the University of California at Santa Cruz (UCSC) Xena platform (https://xena.ucsc.edu/).

The other nine PTC microarray datasets were downloaded from the Gene Expression Omnibus (GEO) database (http://www.ncbi.nlm.nih.gov/geo): GSE3467 (N = 9, T = 9), GSE3678 (N = 7, T = 7), GSE5364 (N = 16, T = 35), GSE27155 (N = 4, T = 51), GSE33630 (N = 45, T = 49), GSE50901 (N = 4, T = 61), GSE53157 (N = 3, T = 15), GSE58545 (N = 18, T = 27), and GSE60542 (N = 30, T = 33).

### Single-sample gene set enrichment analysis (ssGSEA)

The ssGSEA score was used to quantify the enrichment level of 29 immune signatures in each THCA sample (17). To understand the overall situation of immune infiltration and facilitate visualization, we corrected the data as follows. For each ssGSEA score, Xi was replaced with Xi ′ with the equation Xi ′ = (Xi −Xmin)/(Xmax − Xmin), where Xmin and Xmax represent the minimum and maximum values of the ssGSEA score in the THCA sample, respectively (18). The same method was also applied to the other nine GEO datasets.

Additionally, we also measured the infiltration abundance of THCA immune cells through immune cell-specific markers (19). Given that its algorithm and results are highly similar to ssGSEA and are also measurements of immune cell abundance, we present the results in the form of a supplementary graph.

### CIBERSORT

CIBERSORT is a deconvolution algorithm based on RNA-seq data to calculate the composition ratio of 22 immune cells in each sample (20). We performed 1,000 permutations and retained samples of p < 0.05 to ensure the reliability of the results, and the sum of various immune cells was 1. Considering the small sample size of each data set except TCGA after deconvolution, the results of nine GEO data sets were integrated into one validation set.

### Correlation and Clustering of Immune Cells

The Spearman correlation between each pair of cells was calculated and visualized using the R package “corrplot”. Hierarchical agglomerative clustering based on Ward’s linkage and Euclidean distance was used to group immune cells in the PTC microenvironment. An unsupervised clustering algorithm (K-means) was applied to identify immune infiltration patterns and classify patients for further analysis. The results were visualized in the form of an immune network (21).

### Consensus Clustering of PTC Patients based on Immune Signatures

Based on the ssGSEA scores of 29 immune signatures, the R package “ConsensusClusterPlus” was used to perform unsupervised clustering of the THCA samples (22). The cumulative distribution function (CDF), consensus heat map, and principal component analysis (PCA) (23) were applied to evaluate the optimal K (number of groups). We renamed the two groups to the low-immunity group (L-immunity) and to high-immunity group (H-immunity) based on the differences in the ssGSEA scores between the two groups after clustering.

### ESTIMATE

ESTIMATE was used to evaluate the composition of the TME for each THCA sample (24) and included immune score (immune cell infiltration), stromal score (stromal content), ESTIMATE score (stromal-immune comprehensive score) and tumor purity.

### WGCNA Reveals Key Modules and Hub Genes Related to PTC Immunity

First, differentially expressed genes (DEGs) between PTC and adjacent normal tissues in TCGA dataset were identified with the R package “limma” (|log_2_FC| > 1 and adjusted p-value < 0.05).

WGCNA aims to identify coexpressed gene modules, explore associations between gene networks and traits of interest, and discover hub genes in networks (25). DEGs were divided into different gene modules by the R package “WGCNA” (minModuleSize = 30).

The module with the highest absolute module significance was defined as the key module. A gene that satisfies the following conditions was defined as a hub gene.

1. The gene is in the key module.
2. The absolute module membership (MM) > 0.5, which represents Pearson’s correlation between the gene and the module.
3. The absolute gene significance (GS) > 0.5, which represents Pearson’s correlation between the gene and the target traits.

### Analysis of the main mechanism of PTC immunity

#### Functional Enrichment Analysis

The genes in the key module were subjected to Gene Ontology (GO) and Kyoto Encyclopedia of Genes and Genomes (KEGG) enrichment analyses with DAVID 6.8 (https://david.ncifcrf.gov/).

#### Protein-Protein Interaction (PPI) Network Analysis

The hub genes were input into the STRING database (v11.0) (https://string-db.org/) to analyze the interaction network of the hub gene-encoded proteins. The minimum required interaction score was set to 0.4 (medium confidence), and the results were visualized by Cytoscape (v3.7.2).

#### Gene Set Enrichment Analysis (GSEA)

GSEA software (version 4.0.3) (https://www.gsea-msigdb.org/gsea/) was used to explore the main biological function that caused differences between the L-immunity and H-immunity groups. c2.cp.kegg.v6.2.symbols.gmt was set to the reference gene set and permutations=1000 for each analysis. |normalized enrichment score (NES) |≥1.0, p-value ≤ 0.05, and false discovery rate (FDR) q-val ≤ 0.25 were considered statistically significant.

### Statistical Analysis

The *t*-test and Mann-Whitney U test were used for comparisons between two groups. The chi-square test was used to assess differences in clinical parameters between the L-immunity and H-immunity groups. The Pearson and Spearman method was used for correlation analysis. The log-rank method was used to calculate the significant survival *p*-values (progression-free survival (PFS) as the ending indicator). R software (version 3.6.1) and SPSS (v25.0) software were used to analyze the data. R, GraphPad Prism (v8.0) software and Excel were used for data visualization. *p* < 0.05 was considered significant.

## Results

### The Immune System in PTC Showed Overall Enhancement

**Figure 1:**
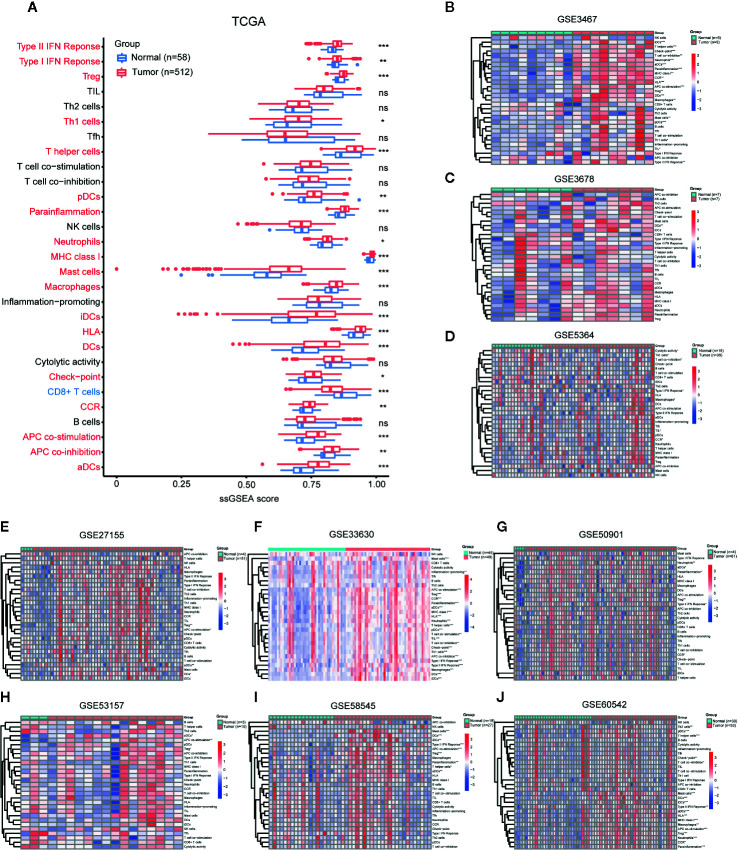
Comparison of immune cell infiltration (abundance) between PTC and normal tissues. Comparison of ssGSEA scores of 29 immune signatures between PTC and normal tissues in **(A)** TCGA, **(B)** GSE3467, **(C)** GSE3678, **(D)** GSE5364, **(E)** GSE27155, **(F)** GSE33630, **(G)** GSE50901, **(H)** GSE53157, **(I)** GSE58545, and **(J)** GSE60542. *p < 0.05, **p < 0.01, ***p < 0.001. ns, not significant.

ssGSEA evaluated 29 immune signatures between PTC and adjacent normal tissues in TCGA (**Figure 1A**), and the results showed that Tregs, Th1 cells, T helper cells, plasmacytoid DCs (pDCs),immmature DCs (iDCs), DCs, activated DCs (aDCs), neutrophils, MCs, and macrophages increased in PTC (*p* < 0.05), while CD8+ T cells decreased in PTC. There were no significant differences in TILs, T_FH_ cells, NK cells, or B cells. Additionally, the type II IFN response, the type I IFN response, parainflammation, MHC class I, HLA, checkpoint, chemokine receptor (CCR) and APC costimulation were elevated in PTC, indicating overall enhancement of the immune system in PTC. The ssGSEA results of the nine GEO validation sets (GSE3467, GSE3678, GSE5364, GSE27155, GSE33630, GSE50901, GSE53157, GSE58545, and GSE60542) also supported the overall enhancement of the immune system in PTC (**Figures 1B–J**).

In addition, immune cell markers in TCGA showed that TPSAB1 (MCs), IL3RA (pDCs), CD68 (macrophages), CD1A (iDCs), and B3GAT1 (NK cells) were overexpressed in PTC. PTPRC (memory T cells), MS4A1 (B cells), IL17A (Th17 cells), CXCR5 (T_FH_ cells), and CD8A (cytotoxic T cells) decreased in PTC (**Figure S1A**).

To summarize the immune cell infiltration results (abundance) of ssGSEA and immune cell markers:

1. Compared with the immune system in normal tissues, the immune system in PTC appears to be enhanced overall.
2. Compared with normal tissues, PTC tissues have an increase in the number of tumor-promoting immune cells, which is particularly significant.
3. Compared with normal tissues, the two algorithms have slightly different evaluations of antitumor immune cells in PTC. The ssGSEA results show that no significant differences in antitumor immune cells, while the immune cell marker results show a downward trend for antitumor immune cell abundance in PTC compared with normal tissues.

### Increased Proportion of Tumor-Promoting Immune Cells in PTC

**Figure 2:**
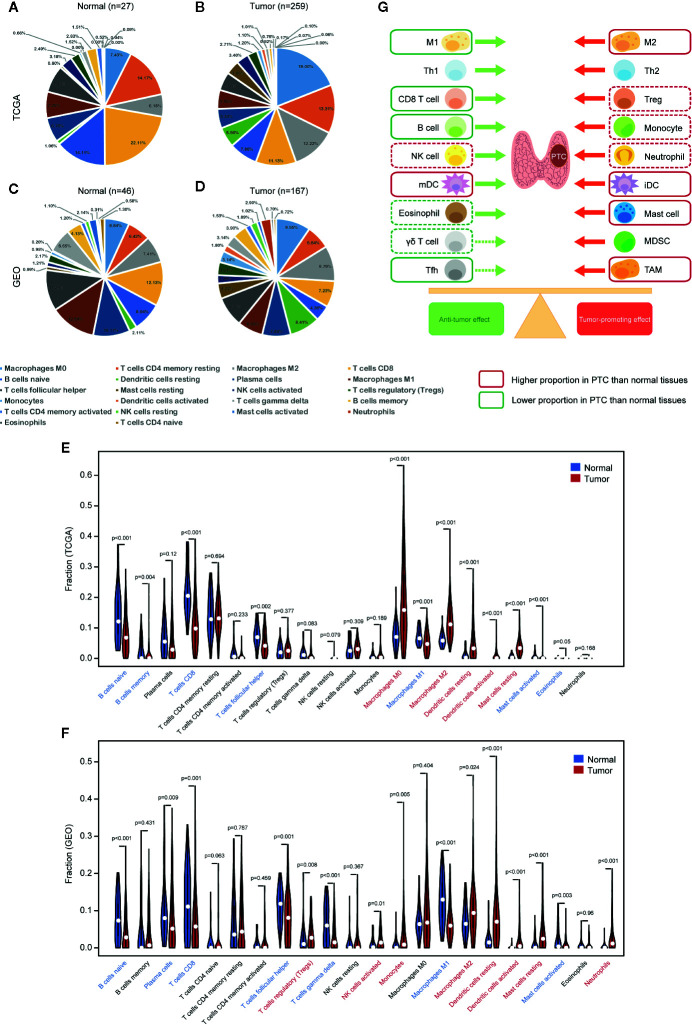
Comparison of immune cell infiltration (proportion) between PTC and normal tissues. Proportions of 22 types of immune cells (CIBERSORT) in **(A)** normal tissues (TCGA), **(B)** PTC tissues (TCGA), **(C)** normal tissues (GEO), and **(D)** PTC tissues (GEO). **(E)** Comparison of immune cell proportions between PTC and normal tissues (TCGA). **(F)** Comparison of immune cell proportions between PTC and normal tissues (GEO). **(G)** Balance chart of the differences in immune cell infiltration between PTC and normal tissues (TCGA and GEO). The dashed box indicates that the difference is significant in TCGA or GEO, while the solid box indicates that the difference is significant in both TCGA and GEO. GEO samples are from nine data sets, including GSE3467, GSE3678, GSE5364, GSE27155, GSE33630, GSE50901, GSE53157, GSE58545, and GSE60542. mDC, mature dendritic cell, iDC, immature dendritic cell.

CIBERSORT was used to calculate the proportion of each of the 22 immune cell types based on RNA-seq data. We performed measurements on the TCGA and GEO datasets (**Figures 2A–D**). The GEO dataset incorporates valid samples from nine datasets, including GSE3467, GSE3678, GSE5364, GSE27155, GSE33630, GSE50901, GSE53157, GSE58545, and GSE60542.

We compared the proportions of immune cells between the PTC and normal tissues in both TCGA and GEO datasets. In TCGA, normal tissues had higher levels of naive B cells, memory B cells, CD8+ T cells, T_FH_ cells, M1 macrophages, activated MCs, and eosinophils than PTC tissues, while PTC tissues had higher levels of M0 macrophages, M2 macrophages, resting DCs, activated DCs, and resting MCs than normal tissues (**Figure 2E**). In GEO, normal tissues had higher levels of naive B cells, plasma cells, CD8+ T cells, T_FH_ cells, T cells gamma delta, M1 macrophages, and activated MCs than PTC tissues, while PTC tissues had higher levels of Tregs, activated NK cells, monocytes, M2 macrophages, resting DCs, activated DCs, resting MCs, and neutrophils than normal tissues (**Figure 2F**). To assist with the understanding of these results, we listed the results in a balance chart (**Figure 2G**). The construction of the balance chart mainly refers to the reviews by Varricchi et al. (14), Galdiero et al. (15) and Ferrari et al. (16) and the literature table that we created (**Table 1**).

**Table 1:** Immune cells in the PTC microenvironment.

Immune cells	Reported data	References
B cells	• In DTCs, B cells were positively correlated with reduced tumor sizes.	(26)
TAMs	• Macrophages are present in the immune environment of PTC and are significantly elevated in the BRAF V600E+ group.	(27)
	• The density of macrophages in PTC tissues is significantly higher than that in normal tissues.	(28, 29),
	• Compared with thyroid goiter and follicular adenoma, PTC tumors have significantly higher TAM densities.	(30)
	• The expression of DCs MCs and macrophages in follicular variant of PTC (FVPTC) is higher than that in adenomas and may be involved in tumor development and invasion.	(31)
	• TAM density is positively correlated with PTC stage, tumor size, lymph node metastasis, and distant metastases.	(24, 29, 30, 32, 33)
	• In diffuse sclerosing variants of PTC (DSV-PTC), M2 TAMs are associated with lymphatic invasion.	(34)
	• CXCL16 signaling mediates the effect of macrophages on PTC tumor cell invasion and changes the macrophage phenotype to M2 TAMs in the PTC microenvironment.	(35)
	• In DTCs, TAMs are positively correlated with reduced tumor sizes and a favorable prognosis.	(26)
	• High TAM density in PTC is associated with poor survival.	(28)
DCs	• DCs are higher in papillary cancer tissues than in normal thyroid tissues, adenomas and other thyroid tumors.	(36–31)
	• Immature DCs (iDCs) in PTC are difficult to induce T cell and NK cell-mediated responses, and they can even suppress immune responses by producing suppressive cytokines such as IL-10 and TGF-β.	(37)
	• Tregs and pDCs together help tumor escape in patients with PTC plus nontoxic goiter (MNG).	(38)
	• The expression of DCs, MCs, and macrophages in FVPTC is higher than that in adenomas and may be involved in tumor development and invasion.	(31)
	• High CD1a+ DC density is associated with improved disease-free survival (DFS) of PTC.	(39)
	• There is no significant correlation between DC density and DFS.	(36)
	• DC immunotherapy can be used in patients with papillary or follicular TC without significant side effects.	(40)
MCs	• MCs are recruited into PTC and promote the proliferation, survival and invasion of cancer cells, thereby promoting the growth and aggressiveness of PTC.	(41)
	• Increased MC density in TC correlates with increased aggressiveness.	(42)
Neutrophils	• NLR is not significantly increased in patients with PTC.	(43)
	• NLR significantly increased in papillary thyroid microcarcinoma (PTMC) and TC patients.	(44)
	• TC cells can recruit neutrophils by releasing CXCL8/IL-8. Additionally, in human TC samples, neutrophil density is related to tumor size and has a potential tumor-promoting effect.	(45)
	• Elevated neutrophils in PTC can predict bilaterality and lymph node metastasis.	(46)
	• Elevated NLR does not appear to be a reliable indicator of DTC progression.	(47)
	• With the increase in the NLR value of PTC, the histopathological characteristics become worse and the clinical behavior becomes more aggressive.	(48–49)
	• There is no significant association between preoperative NLR and prognostic factors in patients with PTC.	(48, 50),
	• Elevated NLR is associated with a high risk of relapse. After treatment, those patients with low stage and good prognosis of DTC were observed to have a significant decrease in NLR.	(51–53),
	• Higher NLR is associated with higher thyroglobulin levels in DTC, which indicates poorer survival.	(54)
NK cells	• Compared with goiter and healthy thyroid, tumor-infiltrating NK cells increase in PTC, while no differences are found in peripheral blood NK cells.	(55–57),
	• In PTC patients, NK cell infiltration is inversely related to the stage of the disease and plays a role of immune surveillance center by killing cancer cells.	(56, 58–60),
T cells	• In PTC, CD8+ T cells kill tumor cells through cytotoxicity and are negatively related to tumor size and lymph node metastasis.	(26, 60–62),
	• In PTC patients, CD8+ T cell infiltration is positively correlated with better prognosis.	(26, 62, 63),
	• A study in patients with DTC showed that the combination of CD8+ cells enrichment and Cox-2 overexpression correlates with the highest risk of disease recurrence.	(64)
	• In human PTC, lymphocyte density is associated with improved overall survival and reduced relapse.	(65, 66),
	• In terms of CD4+ cell frequency, no difference was found between PTC and MNG patients.	(38)
	• In DTC, the extent of tumor-infiltrating CD4+ cells does not seem to predict the patient’s prognosis.	(64)
	• Compared with patients with thyroid adenoma, PTC patients have significantly increased CD4+ CD25+ regulatory T cells in the peripheral blood.	(67)
	• Increased Treg infiltration is positively correlated with advanced PTC.	(58–68)
	• FoxP3 expression in PTC is associated with extrathyroidal invasion and distant metastasis but has nothing to do with overall survival.	(69)
	• Compared with healthy controls, Th17 levels in the peripheral blood and tissue samples of patients with PTC increased, and Th17 cells in the peripheral blood were negatively correlated with tumor size.	(70)

The above table contains the relationship between immune cells and the occurrence, development and prognosis of PTC. Some DTC studies with PTC as the main sample were also included.

In summary, compared with normal thyroid tissues, PTC tissues have a higher proportion of tumor-promoting immune cells and a lower proportion of antitumor immune cells.

### Immune Cell Infiltration Increased During PTC Progression

**Figure 3:**
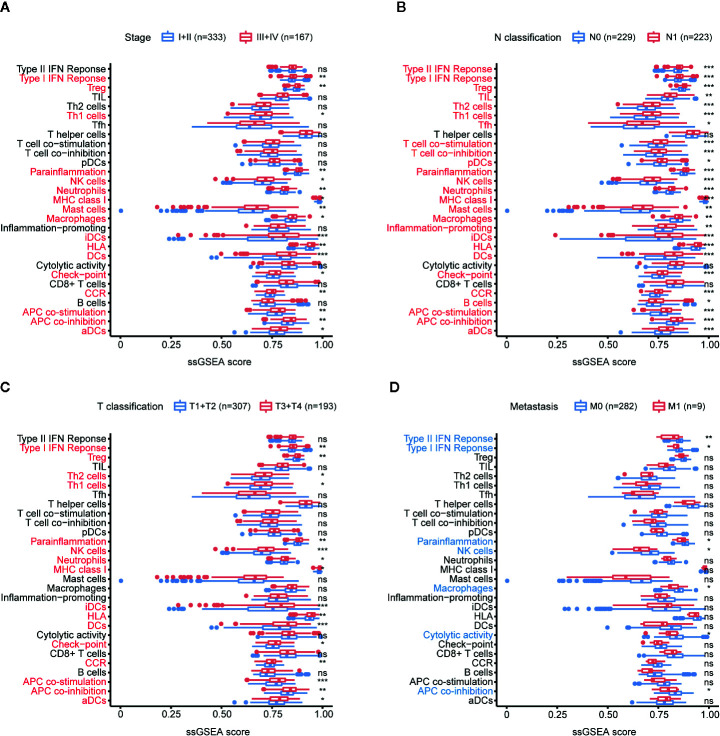
Relationship between immune cell infiltration (abundance) and PTC progression. **(A)** Comparison of the ssGSEA scores of 29 immune signatures between stages I and II PTC patients and stages III and IV PTC patients. **(B)** Comparison of the ssGSEA scores of 29 immune signatures between N0 and N1 PTC patients. **(C)** Comparison of the ssGSEA scores of 29 immune signatures between T1 and T2 PTC patients and T3 and T4 PTC patients. **(D)** Comparison of the ssGSEA scores of 29 immune signatures between PTC patients with and without tumor metastasis. *p < 0.05, **p < 0.01, ***p < 0.001. ns, not significant.

ssGSEA was used to analyze the relationship between PTC progression and immune cell infiltration in TCGA. The results showed that patients with high tumor stage, lymph node metastasis, and larger tumor size had an increased number of immune cells, and the increase in tumor-promoting immune cells was particularly significant, including Th1 cells, macrophages, Tregs, monocytes, neutrophils, iDCs and MCs (**Figures 3A–C**). Patients with tumor metastases had lower immunity levels than patients without metastases (**Figure 3D**).

In addition, the results of immune cell markers also showed that PTC patients with a high tumor stage, lymph node metastasis, and a larger tumor size had an increased number of immune cells, and the increase in tumor-promoting immune cells was particularly significant, including TPSAB1 (MCs), FOXP3 (Tregs), CD1A (iDCs), and neutrophils (CXCR2s) (**Figures S1B–D**).

In summary, with the progression of PTC, immune cell infiltration increased comprehensively.

### The Proportion of Tumor-Promoting Immune Cells Increased During PTC Progression

**Figure 4:**
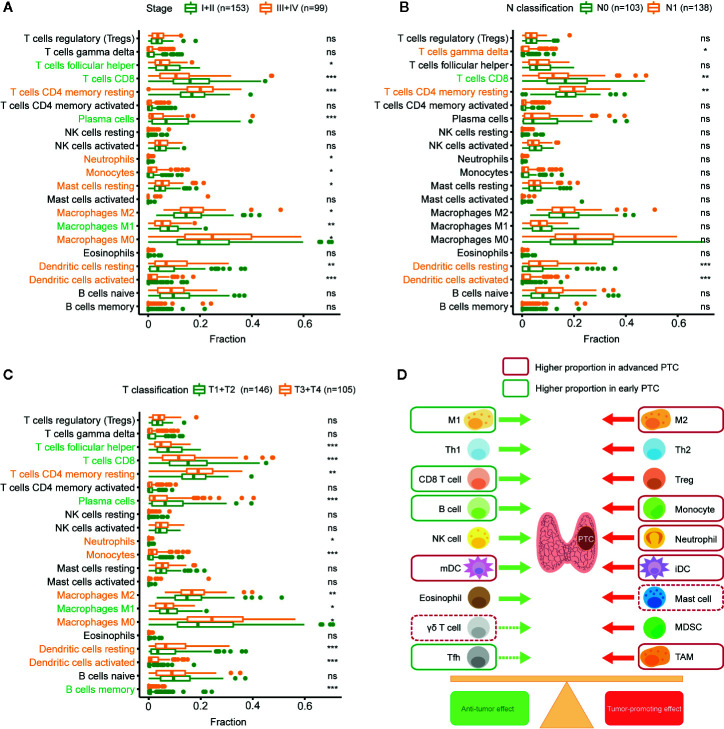
Relationship between immune cell infiltration (proportion) and PTC progression. **(A)** Comparison of immune cell proportion (CIBERSORT) between stages I and II PTC patients and stages III and IV PTC patients. **(B)** Comparison of immune cell proportions between N0 and N1 PTC patients. **(C)** Comparison of immune cell proportions between T1 and T2 PTC patients and T3 and T4 PTC patients. **(D)** Balance chart of the relationship between immune cell infiltration and PTC progression (TCGA). *p < 0.05, **p < 0.01, ***p < 0.001. ns, not significant.

CIBERSORT analyzed the relationship between PTC progression and immune cell infiltration in TCGA. The results show that T_FH_ cells, CD8+ T cells, plasma cells, and M1 macrophages have a higher proportion in stages I and II than in other stages. Resting memory CD4+ T cells, neutrophils, monocytes, resting MCs, M2 macrophages, M0 macrophages, resting DCs, and activated DCs were higher in stages III and IV (**Figure 4A**). CD8+ T cells were higher in N0 PTC, and T cells gamma delta, resting memory CD4+ T cells, resting DCs, and activated DCs were higher in N1 PTC (**Figure 4B**). T_FH_ cells, CD8+ T cells, plasma cells, M1 macrophages, and memory B cells were higher in T1 + T2 PTC. Resting memory CD4+ T cells, neutrophils, monocytes, M2 macrophages, M0 macrophages, resting DCs, and activated DCs were higher in T3 + T4 PTC (**Figure 4C**). These results are further shown in a balance chart (**Figure 4D**).

In summary, compared with early PTC, advanced PTC has a higher proportion of tumor-promoting immune cells and a lower proportion of antitumor immune cells.

### Correlation and Clustering of Immune Cells

**Figure 5:**
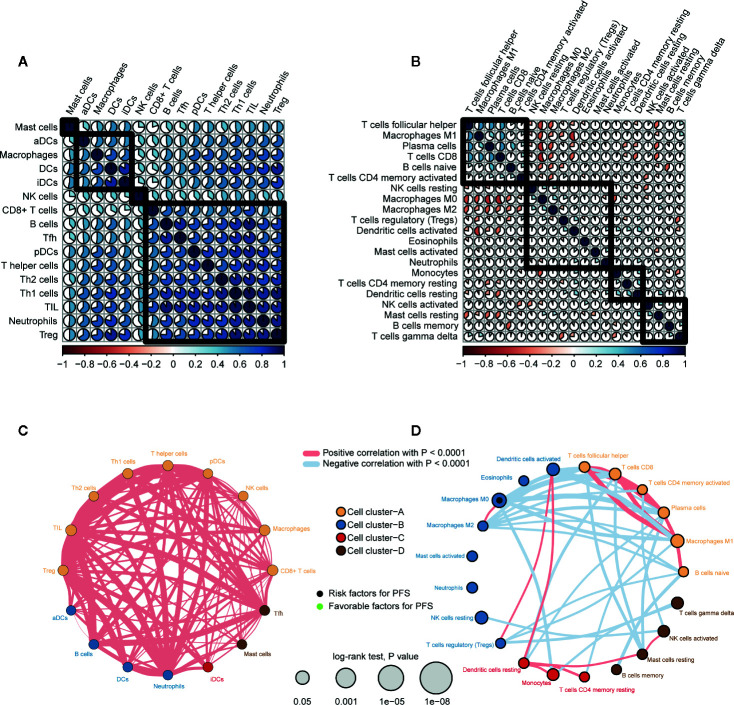
Correlation and clustering of immune cell types in PTC. **(A)** Spearman correlation analysis between immune cells types (abundance, ssGSEA) in PTC. **(B)** Spearman correlation analysis between immune cell types (proportion, CIBERSORT) in PTC. **(C)** Immune network of immune cell types (abundance). The size of each circle represents the effect of each immune cell type on prognosis (PFS). Thicker lines indicate higher Spearman correlations between immune cell types. **(D)** Immune network of immune cell types (proportion).

We performed correlation analysis on immune cells based on ssGSEA (abundance) (**Figure 5A**) and CIBERSORT (proportion) (**Figure 5B**). The ssGSEA results showed a high positive correlation among all immune cells. The CIBERSORT results showed a positive correlation among antitumor immune cells (T_FH_ cells, M1 macrophages, plasma cells, and CD8+ T cells), and these antitumor immune cells were negatively correlated with immune cells with tumor-promoting effects (M0 macrophages, M2 macrophages, Tregs, activated DCs, and resting MCs).

We also clustered immune cells from the perspective of abundance (**Figure 5C**) and proportion (**Figure 5D**) and examined the effect of each immune cell type on prognosis. CIBERSORT results in immune cells (T_FH_ cells, CD8+ T cells, activated memory CD4+ T cells, plasma cells, M1 macrophages, naive B cells) clustered in cell cluster A. In addition, except for M0 macrophages (proportion) (HR = 11.8, log rank *p* = 0.032), the effects of other immune cells on PFS did not reach statistical significance.

In summary, there was a positive correlation among all immune cells. However, antitumor immune cells and tumor-promoting immune cells oppose each other at a proportional level of correlation.

### Consensus Clustering of PTC Patients Based on Immune Signatures

**Figure 6:**
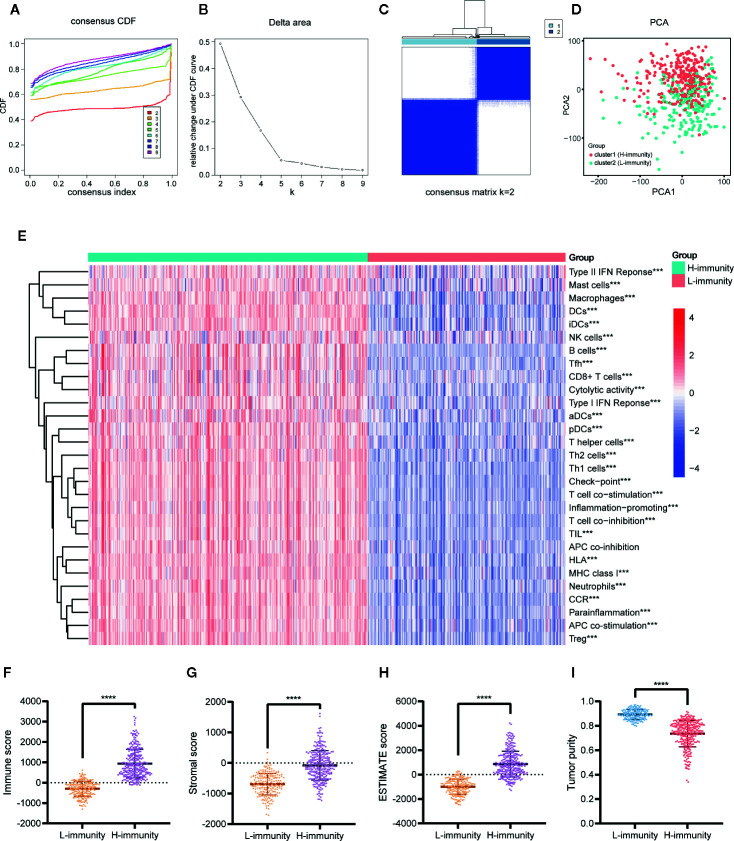
Consensus clustering of PTC patients based on immune signatures. **(A)** Consensus CDF curve (K = 2–9). **(B)** Relative change in area under the CDF curve (K = 2–9). **(C)** Consensus heatmap of K = 2. **(D)** PCA of all genes for the unsupervised clustering results (K = 2). **(E)** Comparison of the ssGSEA scores of 29 immune signatures between the cluster1 (H-immunity) and cluster2 (L-immunity) groups. Comparison of the **(F)** immune scores, **(G)** stromal scores, **(H)** ESTIMATE scores, and **(I)** tumor purity between the L-immunity and H-immunity groups. ***p < 0.001, ****p < 0.001.

Consensus unsupervised clustering was performed on 502 PTC patients based on 29 immune signatures of ssGSEA. We considered the relative change in area under the CDF curve at K = 2 to 9 (**Figures 6A, B**) and found that the consensus heatmaps showed stable partitions when the samples were clustered into two groups (k = 2) (**Figure 6C**). PCA of all genes revealed significant differences between the two groups (**Figure 6D**). We renamed the two groups to the H-immunity (n = 293) and L-immunity groups (n = 209) based on the differences in immune signatures (**Figure 6E**).

The composition of the TME of PTC samples was analyzed by ESTIMATE. The results showed that the H-immunity group had higher immune scores (**Figure 6F**), stromal scores (**Figure 6G**), and ESTIMATE scores (**Figure 6H**) and lower tumor purity (**Figure 6I**) than the L-immunity group.

In summary, we divided PTC patients into L-immunity and H-immunity groups by consensus clustering and verified the excellent discrimination ability of this grouping for all genes, immune signatures, and TME compositions.

### Comparison of Clinical Characteristics Between the H-Immunity and L-Immunity Groups

To understand the effects of immune levels on clinical traits in PTC patients, we compared clinical parameters between the H-immunity and L-immunity groups (**Table 2**). Compared to patients in the L-immunity group, patients in the H-immunity group had an advanced American Joint Committee on Cancer (AJCC) stage, advanced T classification, lymph node metastasis, higher tall-cell PTC and lower follicular PTC proportions, more BRAF mutations and fewer RAS mutations.

**Table 2:** Comparison of the clinical parameters between the H-immunity and L-immunity groups in PTC.

Clinical parameters	H-immunity (n = 293, %)	L-immunity (n = 209, %)	*p*-value
**Age (y)**			
<55	199 (67.9)	136 (65.1)	0.505
≥55	94 (32.1)	73 (34.9)	
**Gender**			
Female	215 (73.4)	152 (72.7)	0.871
Male	78 (26.6)	57 (27.3)	
**Stage**			
I	161 (54.9)	120 (58.0)	0.001
II	19 (6.5)	33 (15.9)	
III	76 (25.9)	36 (17.4)	
IV	37 (12.6)	18 (8.7)	
NA	0	2	
**Metastasis**			
M0	184 (97.9)	98 (95.1)	0.199
M1	4 (2.1)	5 (4.9)	
NA	105	106	
**N classification**			
N0	123 (43.9)	106 (61.6)	<0.001
N1	157 (56.1)	66 (38.4)	
NA	13	37	
**T classification**			
T1	87 (29.8)	56 (26.9)	0.018
T2	80 (27.4)	84 (40.4)	
T3	109 (37.3)	61 (29.3)	
T4	16 (5.5)	7 (3.4)	
NA	1	1	
**Radiation therapy**			
Yes	175 (62.7)	130 (64.0)	0.767
No	104 (37.3)	73 (36.0)	
NA	14	6	
**Pathological type**			
Classical	231 (78.8)	125 (59.8)	<0.001
Follicular	23 (7.8)	78 (37.3)	
Tall cell	35 (11.9)	1 (0.5)	
Other	4 (1.4)	5 (2.4)	
**BRAF**			
Wild-type	63 (22.4)	132 (65.7)	<0.001
Mutated	218 (77.6)	69 (34.3)	
NA	12	8	
**RAS**			
Wild-type	275 (97.9)	147 (73.1)	<0.001
Mutated	6 (2.1)	54 (26.9)	
NA	12	8	

### WGCNA Screening Key Modules and Hub Genes Related to PTCImmunity

**Figure 7:**
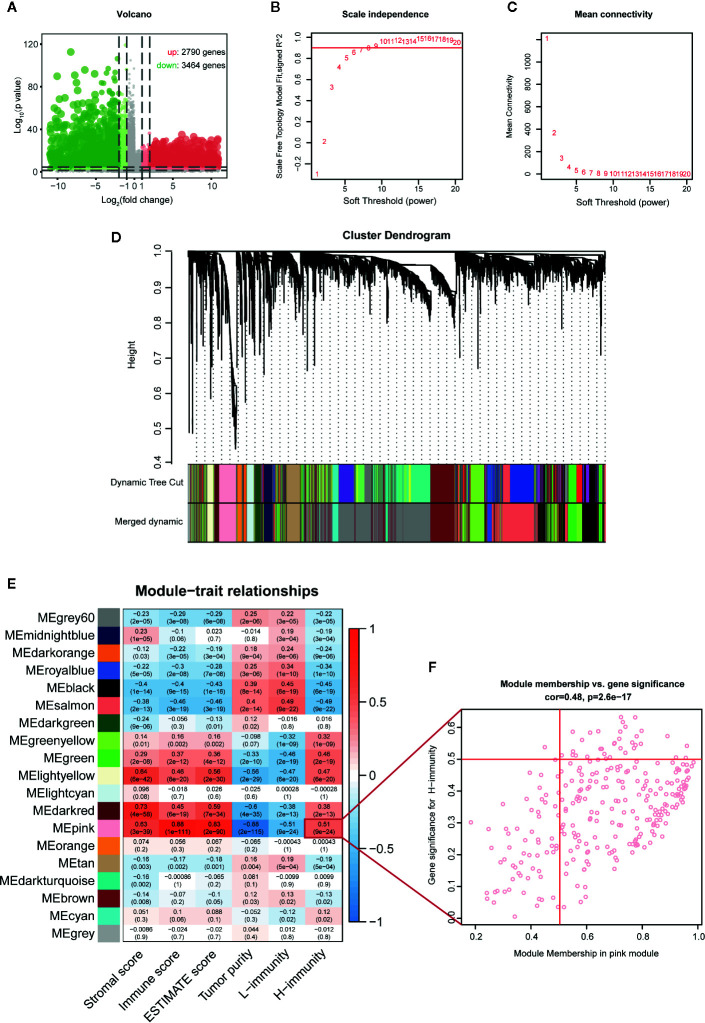
WGCNA screening key modules and hub genes related to PTC immunity. **(A)** Volcano map of DEGs between PTC and adjacent tissues (TCGA). **(B)** Calculation of the scale-free fit index of various soft-thresholding powers (β). **(C)** Analysis of the mean connectivity of various soft-thresholding powers (β). **(D)** Clustering dendrogram of 502 PTC patients, where 6254 DEGs were clustered based on the dissimilarity measure (1-TOM) and divided into 19 modules. **(E)** Correlation heatmap between module eigengenes and immune traits; the modules with high relevance to the H-immunity group are the key modules of mechanism research. **(F)** Scatter plot of the key module eigengenes (pink).

To construct a weighted coexpression network and screen for modules and genes related to PTC immunity, 6254 DEGs between PTC (n = 512) and normal tissues (n = 58) in TCGA were selected for subsequent WGCNA (502 patients with complete clinical information were selected) (**Figure 7A**). After a series of adjustments to the WGCNA parameters, the DEGs were divided into 19 modules by average linkage hierarchical clustering (**Figures 7B–D**). The correlation heatmap showed that the pink module, which contains 276 genes, had the highest correlation with the H-immunity group (Pearson’s correlation coefficient = 0.51, *p* < 0.001) (**Figure 7E**). The 34 genes in the pink module with MM > 0.5 and GS > 0.5 were defined as hub genes (**Figure 7F**).

### Analysis of the Main Mechanism of PTC Immunity

**Figure 8:**
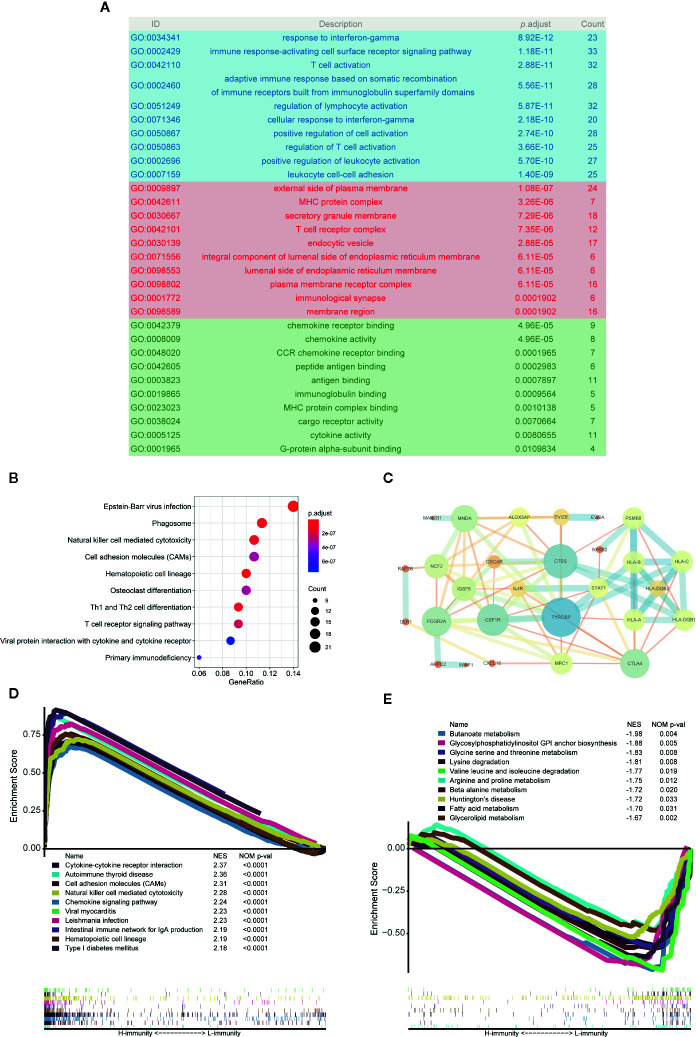
Analysis of the main mechanism of PTC immunity. **(A)** GO analysis of 276 pink module eigengenes; the blue, red and green areas represent biological processes, cellular components, and molecular functions, respectively. **(B)** KEGG analysis of pink module eigengenes: the top 10 significantly enriched pathways are shown. **(C)** Protein-protein interactions (PPIs) of hub genes. **(D)** Top 10 enriched pathways in the H-immunity group based on GSEA. **(E)** Top 10 enriched pathways in the L-immunity group based on GSEA.

A total of 276 pink module eigengenes were used for functional and pathway enrichment analyses to explore potential mechanisms that cause differences in the immune microenvironment between PTCs. “Response to interferon-gamma”, “external side of plasma membrane”, and “chemokine receptor binding” were the most common GO terms for biological processes, cellular components, and molecular functions, respectively (**Figure 8A**). “Epstein-Barr virus infection” was the most significantly enriched pathway in KEGG analysis (*p*-adjust < 0.001) (**Figure 8B**). Additionally, we constructed a PPI network with the 34 hub genes and found that *TYROBP*, *CTLA4*, *CSF1R*, *CTSS*, and *HLAs* play key roles (**Figure 8C**).

GSEA was also used to identify the mechanism and functional differences between the L-immunity and H-immunity groups. The H-immunity group was enriched in the following terms: cytokine-cytokine receptor interaction, autoimmune thyroid disease, cell adhesion molecules (CAMs), natural killer cell-mediated cytotoxicity, chemokine signaling pathway, viral myocarditis, Leishmania infection, intestinal immune network for IgA production, hematopoietic cell lineage, and type I diabetes mellitus (**Figure 8D**). Conversely, the L-immunity group was enriched in the following metabolism-related pathways: butanoate metabolism; glycosylphosphatidylinositol (GPI) anchor biosynthesis; glycine, serine, and threonine metabolism; lysine degradation; valine, leucine, and isoleucine degradation; arginine and proline metabolism; beta alanine metabolism; Huntington’s disease; fatty acid metabolism; and glycerolipid metabolism (**Figure 8E**).

## Discussion

Our findings showed that during the occurrence and development of PTC, the immune system is enhanced; moreover, M2 macrophages, Tregs, monocytes, neutrophils, DCs, MCs, and M0 macrophages play a tumor-promoting role, and their abundances and proportions are significantly increased. M1 macrophages, CD8+ T cells, B cells, NK cells, and T_FH_ cells (eosinophils, γδ T cells, and Th17 cells with weak supporting evidence) exert an antitumor effect, and the proportions decreased.

PTC is subdivided into “inflammatory” tumors (71). Extensive evidence shows that autoimmunity and inflammation are risk factors for TC (72). Increasing evidence also suggests that cancer-related inflammation may be a useful target for new diagnostic and therapeutic strategies in TCs (73). Therefore, to evaluate the specific pattern of immune cells involved in PTC, including not only its phenotype but also its function, it is essential to understand the immunological characteristics of PTC. Many major advances have been made in the study of immune infiltration in PTC, especially in major immune cells (B cells, TAMs, NK cells, neutrophils, DCs, MCs, CD8+ T cells and Tregs) (14–16). A considerable part of our research is repeating, verifying, and integrating previously reported results of immune cell phenotypes and functions in PTC. We used ssGSEA, immune cell marker and CIBERSORT to evaluate the infiltration level of immune cells in 10 datasets (a total of 799 PTC and 194 normal samples) from the perspective of abundance and proportion, respectively. Furthermore, it is essential to ensure that the results of the analysis are highly reliable. We also analyzed subtypes of major immune cells and immune cells that lacked attention (such as M1 macrophages, M2 macrophages, Th1, Th2, pDCs, iDCs, eosinophils, monocytes, T_FH_ cells, T cells gamma delta, and Th17 cells) This will help researchers comprehensively understand the PTC immune microenvironment. Our research supports the evidence that M2 macrophages, Tregs, monocytes, neutrophils, DCs, MCs, and TAMs play a tumor-promoting role, while M1 macrophages, CD8+ T cells, B cells, NK cells, and T_FH_ cells (eosinophils, γδ T cells, and Th17 cells with weak supporting evidence) exert an antitumor effect. Among them, related reports of the phenotype and function of monocytes, γδ T cells, T_FH_ cells, and eosinophils in PTC patients are still lacking. Inflammation of monocyte recruitment and activation is associated with the development of TC in a mouse model (74). IL-17-producing γδT17 cells play a decisive role in the chemotherapy-induced anticancer immune response (75). T_FH_ cells can regulate antibody production by B cells (76); the frequency of T_FH_ cells increases in patients with autoimmune thyroid disease (AITD), and a significant number of T_FH_ cells are also detected in the thyroid tissues of patients with Hashimoto’s thyroiditis (77). The above evidence also supports our conclusions about the function of immune cells.

Our research shows that compared with the immune level in normal tissues, the immune level in PTC tissues is generally higher, and the increase in tumor-promoting immune cells is particularly significant. The proportion of immune cells in the TME is also favoring tumor-promoting immune cells. The elevation of TAMs, DCs, MCs, neutrophils, NK cells, and Tregs in PTC has been reported (28–31, 36, 38, 41, 45, 55, 78). Our study found that these tumor-promoting immune cells were recruited in large numbers in PTC and exhibited a tumor-promoting immune microenvironment phenotype. This indicates that PTCs manipulate partial immune cells such as M2 macrophages, Tregs, monocytes, neutrophils, DCs, MCs, and M0 macrophages to fight against the body’s immune response to protect themselves, which is also called “sabotage” (13). Eventually, the tumor evaded immune surveillance and escaped the immune system.

In additional research, we found that immune cell infiltration increased during tumor progression, and the increase in tumor-promoting immune cells was particularly significant. The proportion of immune cells in the TME was further tilted towards tumor-promoting immune cells. Previous mainstream studies have shown that TAMs, DCs, MCs, neutrophils, and Tregs are positively correlated with PTC progression (24, 29–34, 38, 41, 42, 46, 48, 49, 51, 58, 61, 68, 69, 79), and B cells, CD8+ T cells, NK cells, and iDCs are negatively correlated with PTC progression (26, 37, 56, 58–62). Unlike previous studies, our research found that both the abundance and proportion of tumor-promoting immune cells were positively correlated with PTC progression, while the proportion of antitumor immune cells during the progression of PTC decreased (CIBERSORT), and the abundance increased (ssGSEA) or did not significantly change (immune cell marker). These results show that in escalating immune confrontation, immune escape is aggravated, and the tumor’s ability to fight the immune response of the body is further strengthened by recruiting immunosuppressive cells. Eventually, the dynamic balance between antitumor and tumor-promoting immune cells in the original immune system has allowed the irreversible development of tumors. The positive correlations among the H-immunity group, tumor stage, lymph node metastasis, and tumor size also support the above hypothesis.

Our analysis of the correlation of the abundances of immune cells in PTC showed a high positive correlation among all examined cells, indicating that the immune system as a whole has a high degree of consistency. However, antitumor immune cells and tumor-promoting immune cells oppose each other at a proportional level of correlation. Our clustering results on immune cells also show the opposition between immune cells that exert procancer and anticancer effects. We speculate that in the process of tumor progression, the confrontation between tumor-killing immune cells and tumor-controlled immunosuppressive cells in the human immune system continues to escalate, and eventually, the proportion of tumor-promoting immune cells in the TME will have an irreversible advantage. This indicates that the tumor cells escaped the body’s immune surveillance by “sabotage” and achieved immune escape. Except for M0 macrophages (proportion) in our prognosis studies, other immune cells did not reach significant levels. Previous studies in PTC have generally shown that TAMs and neutrophils are associated with a worse prognosis (28, 51–54), while CD8+ T cells and DCs are associated with a better prognosis (26, 39, 62, 63). In fact, the roles of various immune cells in prognosis are still controversial (26, 64), and there are many negative results (36, 48, 50, 64, 70). Considering the positive correlation between immune cell infiltration and tumor progression, we suspect that the insignificant prognosis of immune cells may be related to the lower number of outcomes, such as death, recurrence and metastasis of PTC.

We divided patients into L-immunity and H-immunity groups by consensus clustering and verified the excellent discrimination ability of this grouping for all genes, immune signatures, and TME composition. Compared to patients in the L-immunity group, patients in the H-immunity group showed a more advanced stage, larger tumor size, more lymph node metastasis, higher tall-cell PTC proportions, lower follicular PTC proportions, more BRAF mutations and fewer RAS mutations. The BRAF V600E mutation is related to the immunosuppressive mechanism of PTC; compared to the ratio in wild-type BRAF PTC, the CD8+/FoxP3+ ratio in BRAF V600E mutant PTC was significantly reduced, and the proportion of M2 type TAMs was increased (27, 80, 81). We speculate that somatic mutations in genes such as BRAF and RAS in PTC may initiate the changes in the tumor immune microenvironment.

WGCNA and GSEA were used to explore the underlying mechanisms that cause PTC immune differences, and a number of significantly different immune-related functions and pathways were found. What caught our attention was that Epstein-Barr virus (EBV) infection was the most significantly enriched KEGG pathway. Despite some controversy, many studies have pointed out that EBV is highly expressed in patients with TC and is associated with increasing development of thyroid tumors (82–85). Studies related to nasopharyngeal carcinoma (NPC), Burkitt lymphoma (BL) and gastric cancer have found that EBV can evade host immune recognition and latent infection in B lymphocytes, can epigenetically suppress host tumor suppressor genes, and can provide a potential “hit and run” mechanism for viral carcinogenesis (86–88). We speculate that EBV may play an important role in the change in the PTC immune microenvironment.

However, this study has some limitations. First, ssGSEA, immune cell markers, and CIBERSORT are algorithms based on RNA-seq data, and the abundant results of ssGSEA and immune cell markers are inconsistent in some antitumor immune cells. We have shown their respective results and common conclusions. Second, the research on the mechanism that causes immune differences was based on the inference of several algorithms and has not been experimentally verified.

In the next study, single-cell analysis of immune cells around and within tumors will be an important direction for PTC immune research to elucidate the function of immune cells in the PTC TME.

In conclusion, our research shows that M2 macrophages, Tregs, monocytes, neutrophils, DCs, MCs, and M0 macrophages play a tumor-promoting role in PTC, while M1 macrophages, CD8+ T cells, B cells, NK cells, T_FH_ cells (eosinophils, γδ T cells, and Th17 cells with weak supporting evidence) play an antitumor role. During the occurrence and development of PTC, the overall immune level increased, and the abundance and proportion of tumor-promoting immune cells significantly increased. We speculate that EBV may play an important role in changing the immune microenvironment of PTC.

## Data Availability Statement

The original contributions presented in the study are included in the article/**Supplementary Materials**, further inquiries can be directed to the corresponding author/s.

## Ethics Statement

Ethical review and approval was not required for the study on human participants in accordance with the local legislation and institutional requirements. Written informed consent for participation was not required for this study in accordance with the national legislation and the institutional requirements.

## Author Contributions

ZX designed the analytical strategies, performed data analyses, and wrote the manuscript. YL, XL, YZH, SW, SYW, JS, and YCH performed data analyses. JZ conceived the research and wrote the manuscript. All authors contributed to the article and approved the submitted version.

## Funding

This research was supported by the National Natural Science Foundation of China (81600602).

## Conflict of Interest

The authors declare that the research was conducted in the absence of any commercial or financial relationships that could be construed as a potential conflict of interest.

## Supplementary Material

Supplementary Figure 1 Changes in immune cell infiltration (immune cell-specific markers) during the occurrence and development of PTC. **(A)** Comparison of immune cell abundance between PTC and normal tissues. **(B)** Comparison of immune cell abundance between stages I and II PTC patients and stages III and IV PTC patients. **(C)** Comparison of immune cell abundance between N0 and N1 PTC patients. **(D)** Comparison of immune cell abundance between T1 and T2 PTC patients and T3 and T4 PTC patients.

## Abbreviations

aDCs, activated dendritic cells; AJCC, American Joint Committee on Cancer; BL, Burkitt lymphoma; CAMs, cell adhesion molecules; CCR, chemokine receptor; CDF, cumulative distribution function; DCs, dendritic cells; DEGs, differentially expressed genes; DTC, differentiated thyroid carcinoma; EBV, Epstein-Barr virus; FDR, false discovery rate; GEO, Gene Expression Omnibus; GO, Gene Ontology; GPI, glycosylphosphatidylinositol; GSEA, gene set enrichment analysis; iDCs, immmature dendritic cells; KEGG, Kyoto Encyclopedia of Genes and Genomes; MCs, mast cells; mDCs, mature dendritic cells; MDSCs, myeloid-derived suppressor cells; MHC-I, major histocompatibility complex class I; NES, normalized enrichment score; NPC, nasopharyngeal carcinoma; PCA, principal component analysis; pDCs, plasmacytoid dendritic cells; PFS, progression-free survival; PPI, Protein-protein interaction; PTC, Papillary thyroid carcinoma; ssGSEA, single-sample gene set enrichment analysis; TAMs, macrophages; TC, Thyroid cancer; TCGA, The Cancer Genome Atlas; TFH cell, T follicular helper cell; THCA, thyroid carcinoma in The Cancer Genome Atlas; TME, tumor microenvironment; UCSC, University of California at Santa Cruz; WGCNA, Weighted gene coexpression network analysis.
