# Supplementary figures and images for: Immune Cell Confrontation in the Papillary Thyroid Carcinoma Microenvironment
Source: Front Endocrinol (Lausanne). 2020 Oct 22;11:570604. doi: 10.3389/fendo.2020.570604 (PMC7642595; doi:10.3389/fendo.2020.570604)

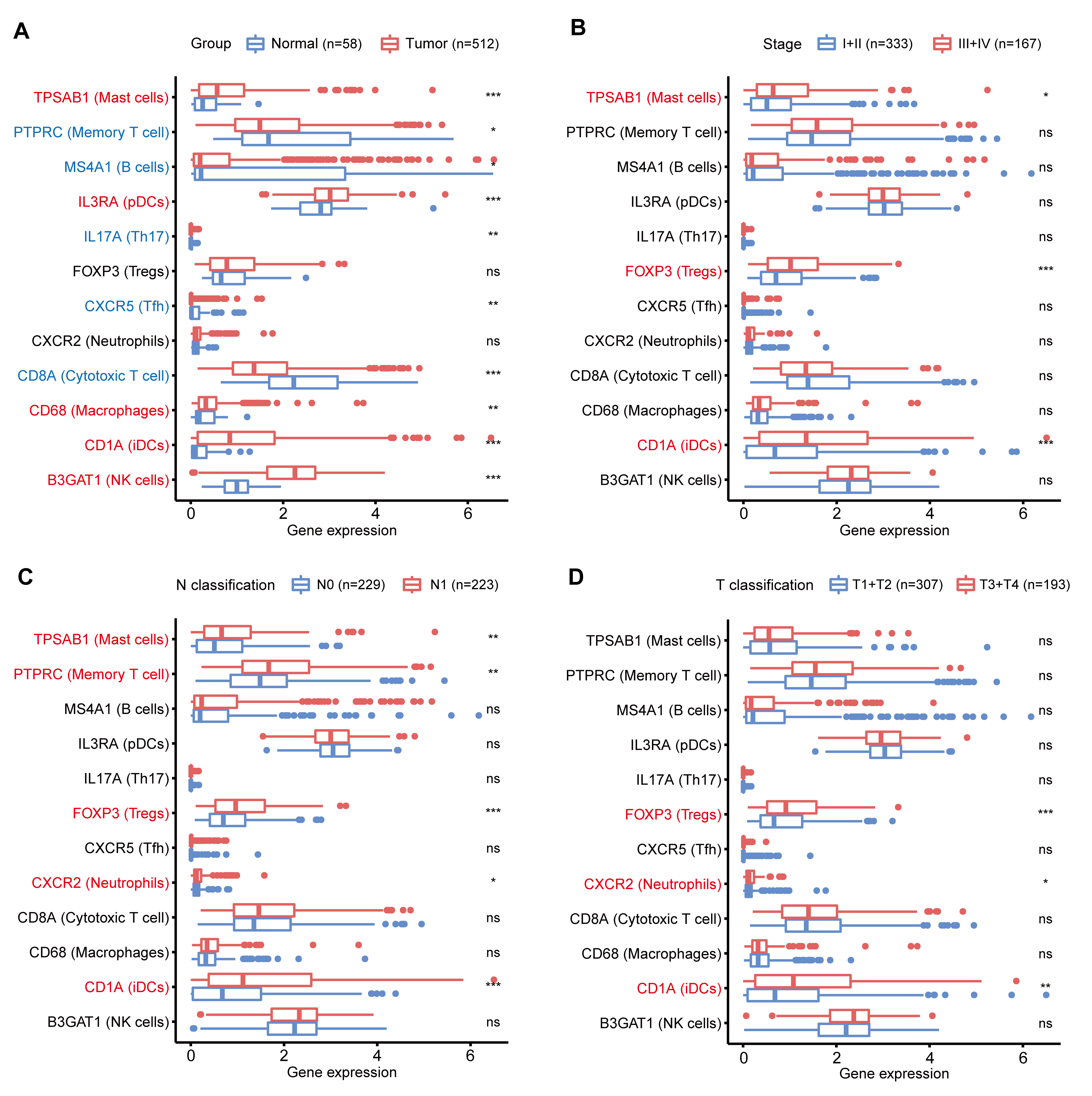

Supplement: Supplementary Figure 1 — Changes in immune cell infiltration (immune cell-specific markers) during the occurrence and development of PTC. (A) Comparison of immune cell abundance between PTC and normal tissues. (B) Comparison of immune cell abundance between stages I and II PTC patients and stages III and IV PTC patients. (C) Comparison of immune cell abundance between N0 and N1 PTC patients. (D) Comparison of immune cell abundance between T1 and T2 PTC patients and T3 and T4 PTC patients. [file Image_1.tif]
